# Expression of Hepatitis B Virus Surface Antigen Containing Y100C Variant Frequently Detected in Occult HBV Infection

**DOI:** 10.1155/2011/695859

**Published:** 2011-02-06

**Authors:** Francisco C. A. Mello, Nora Martel, Selma A. Gomes, Natalia M. Araujo

**Affiliations:** ^1^Laboratory of Molecular Virology, Oswaldo Cruz Institute, FIOCRUZ, Avenida Brasil 4365, 21045-900 Rio de Janeiro, RJ, Brazil; ^2^INSERM U871, 151 Cours Albert Thomas, 69424 Lyon, France

## Abstract

Small hepatitis B virus surface protein (S-HBsAg) variant Y100C has been associated with HBsAg-negative phenotype. To determine whether Y100C substitution yields impaired HBsAg or small amounts of HBsAg that may reduce HBsAg detection by commercial anti-HBsAg antibodies, two eukaryotic expression plasmids, one containing a wild-type S and the other an S gene from a Y100C variant, were constructed and their levels of HBsAg compared by ELISA after transfection of HuH7 cells. Unexpectedly, the extracellular HBsAg levels detected with Y100C plasmid were higher than those observed with the wild-type plasmid, but without statistical significance. We concluded that the Y100C substitution alone did not play a role in reducing HBsAg amounts or HBsAg affinity by commercial ELISA assay. Further studies on *in vitro* replication fitness with the complete genome of HBV isolates displaying or not Y100C substitution may elucidate whether this mutation affects HBV replication and consequently HBsAg production.

## 1. Introduction

Hepatitis B virus (HBV) infection still constitutes a major problem of public health since 360 million people worldwide are chronically infected [1] and exposed to a progressive disease that may lead to liver cirrhosis and hepatocellular carcinoma (HCC) [2]. HBV surface antigen (HBsAg) is the established serological marker for the diagnosis of acute or chronic HBV infection, and the absence of HBsAg in serum has been used as a surrogate marker for the absence of DNA and active viral replication. However, in the last decade, the development of highly sensitive molecular biology techniques allowed the detection of low levels of HBV DNA in serum samples and/or liver of individuals negative for HBsAg [3, 4]. This peculiar form of chronic viral infection has been termed occult HBV infection [5–7]. 

Different mechanisms may be responsible for the absence of HBsAg in occult infection, which includes (i) low rate of HBV replication due to host's immune response or coinfection with other infectious agents [8–10], (ii) association of HBsAg to anti-HBs resulting in the formation of immune complexes that reduce the circulation of free antigen [5], or (iii) mutations that inhibit HBsAg expression [11] or change HBsAg antigenicity, thus preventing detection by commercial assays [6, 12–16].

Tyrosine (Y) to cysteine (C) substitution in HBsAg residue 100 (Y100C) has been associated with HBsAg-negative phenotype in blood donors from Venezuela [17] and Spain [18], and is frequently found in cases of occult HBV infection in Brazil [12, 19, 20]. Additionally, this substitution has also been described in one Argentinean patient exhibiting cocirculation of HBsAg and anti-HBs antibodies [21]. 

In this study, the role of Y100C substitution in reducing amount of HBsAg or changing HBsAg affinity by commercial antibodies was investigated by comparison of HBsAg levels obtained from recombinant plasmids with or without Y100C substitution.

## 2. Materials and Methods

### 2.1. Construction of HBsAg Expression Plasmids

Two different HBV isolates previously characterized in our laboratory as belonging to HBV subgenotype A1 were used for plasmid constructions. One isolate had deduced amino acid sequence for small S gene that was identical to a consensus sequence of wild-type HBV subgenotype A1. Consensus was originated from an alignment of 100 HBV complete genome sequences from HBV subgenotype A1 available in GenBank. The other has a unique Y100C variation in its amino acid sequence. These two viral strains were used to construct two recombinant expression plasmids under CMV promoter, representing a wild-type and a Y100C variant of HBV S gene (named pcDNA3-SA1 and pcDNA3-Y100C, resp.). Briefly, HBV DNAs were phenol/chloroform extracted from 250 *μ*L of serum as previously described [22]. The region coding for S-HBsAg was amplified by seminested PCR assay. The first round of amplification was performed with PS1 and S2 primers and the second round of amplification with S1 and S2 primers, as previously described [12]. S gene PCR products were cloned into the pCR4 cloning vector (TOPO TA Cloning Kit for Sequencing, Invitrogen, San Diego, CA) and subcloned into the EcoRI polylinker region of the mammalian cell expression vector pcDNA3 (Invitrogen) as previously described [12]. S-HBsAg expression plasmids were purified using S.N.A.P. Midiprep kit (Invitrogen). HBV nucleotide sequences were determined using BigDye Terminator kit (Applied Biosystems, CA, USA). Sequencing reactions were analyzed on an ABI 3730 automated sequencer (Applied Biosystems). Bioinformatics analysis of the sequences was performed using MEGA version 3.1 software [23]. Wild-type and Y100C variant sequences determined have been deposited in the GenBank database, accession numbers HQ840709 and EF690524, respectively.

### 2.2. Cell Culture and Transfection Assays

Transient transfection assays were performed using human hepatoma HuH7 cells maintained in Dulbecco's modified Eagle medium supplemented with 10% fetal bovine serum. Cells were plated at a density of 5 × 10^5^ cells per well of 6-well plates and transfected with 2 *μ*g of HBsAg expressing plasmid using FuGENE 6 (Roche Diagnostics, Mannheim, Germany), according to the manufacturer's instructions. Three independent transfection assays were performed using three different plasmid preparations of each construct. Culture supernatants were collected on day 5 after transfection and clarified by centrifugation at 1,500 g for 5 min. To evaluate HBsAg levels inside cells, cell monolayers were washed twice with phosphate buffered saline (PBS), scraped with 1 mL of PBS and centrifuged at 2,500 g for 5 min. Cell pellets were frozen/thawed three times and resuspended in 0.4 mL of PBS. Medium and cell extracts were tested for the presence of HBsAg by a commercial immunoassay (BioELISA HBsAg colour, Biokit, Barcelona, Spain) after a serial dilution of 10 times in the medium. Mock-transfected HuH7 cells were used as negative control. HBsAg were quantified using an international panel of reference sera containing different concentrations of HBsAg (Boston Biomedica Inc, USA). HBsAg concentrations of the panel are expressed in ng/mL and in international units (IU). The detection limit of the assay is about 0.25 ng/mL corresponding to 0.05 IU. HBsAg levels produced by both plasmids were compared using Mann-Whitney U test with the Statistical Package for Social Sciences (SPSS, Chicago, IL, USA), version 17.0. Significance was set at a *P* value of less than .05.

## 3. Results

**Figure 1:**
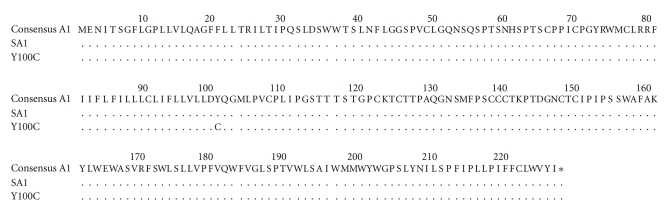
Comparison of HBsAg sequences, deduced from recombinant plasmids representing a wild-type (pcDNA3-SA1) and a mutated (pcDNA3-Y100C) HBV with a consensus sequence of HBV subgenotype A1. Consensus was originated from an alignment of 100 HBV complete genome sequences from HBV subgenotype A1 available in GenBank.

Nucleotide sequencing of HBV S gene inserted in recombinant expression plasmids pcDNA3-SA1 and pcDNA3-Y100C had confirmed the high similarity between both sequences. A total of four nucleotide mismatches (T36G, A299G, T432C, and C666T) between pcDNA3-Y100C and pcDNA3-SA1 was found along the 681-bp fragment of S gene (not shown). The A299G is the only nonsynonymous substitution changing amino acid Y to C in HBsAg residue 100 of small S protein (Figure 1).

**Figure 2:**
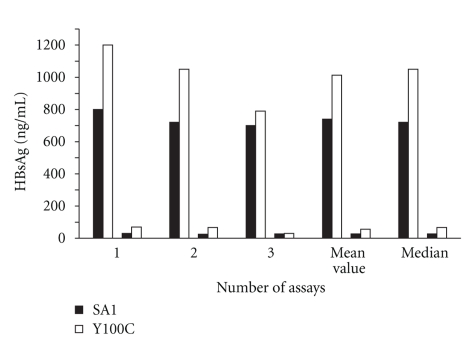
Detection of extracellular (upper columns) and intracellular (lower columns) levels of HBsAg by ELISA after transfection of HuH7 cells. Black columns: SA1; white columns: Y100C variant. 1, 2, 3: HBsAg levels obtained in each transfection assay.

HBsAg levels of medium and cells extracts of pcDNA3-SA1 and pcDNA3-Y100C constructs, tested in three independent transfection assays, were shown in Figure 2. HBsAg levels detected in medium of transfected cells with pcDNA3-SA1 ranged from 700 to 800 ng/mL (mean and median values of 740 ng/mL (corresponding to 144 IU) and 720 ng/mL (143 IU), resp.), while variation in pcDNA3-Y100C construct ranged from 790 to 1200 ng/mL (mean and median values of 1.013 ng/mL (230 IU) and 1.050 ng/mL (250 IU), resp.). The difference between the amount of HBsAg produced by pcDNA3-SA1 and pcDNA3-Y100C was not significant (*P* = .13). Overall, the mean values of extracellular HBsAg of pcDNA3-Y100C plasmid were 1.5-fold higher than those observed when the wild-type plasmid was transfected. Values of HBsAg detected in cell extracts were much lower than those in medium with both plasmids (pcDNA3-SA1 and pcDNA3-Y100C), indicating that S-HBsAg was secreted well in both cases. HBsAg levels detected in extracellular medium were about 27-fold and 15-fold higher than that of cells extracts with pcDNA3-SA1 and pcDNA3-Y100C plasmids, respectively.

## 4. Discussion

Occult hepatitis B infection has been the subject of several studies due to different clinical contexts associated with it such as the transmission of HBV in blood transfusion and organ transplantation, virological and clinical reactivation, negative influence on the clinical outcome of other chronic liver diseases, and development of hepatocellular carcinoma [6]. The Y100C variation of the small S protein has been frequently detected in the absence of HBsAg and has been statistically associated with occult infection in studies conducted in Venezuela [17] and in Brazil [12, 19, 20]. A variety of explanations for persistence of HBV DNA in HBsAg-negative individuals have been proposed, including the occurrence of amino acid substitutions in the HBsAg. Several HBsAg variants with reduced reactivity against anti-HBs antibodies have been characterized, most of them with specific mutations in the a determinant of HBsAg [24–28]. Viruses with G145R replacement have been almost invariably isolated as a major escape mutant after HBV vaccination [16, 25, 27, 29]. Besides mutations leading to reduced reactivity against anti-HBs antibodies, some mutations may cause intracellular retention of HBsAg and might play roles in the several types of situations in which HBV DNA is detected in the absence of HBsAg [13, 30, 31]. In the present study, we evaluated if Y100C substitution alone may influence the secretion pattern of HBsAg and/or may modify reactivity against commercial antibodies. Previous studies have shown that the presence of charged or polar amino acids in a transmembrane (TM) segment had a crucial role in endoplasmic reticulum (ER) retention [32, 33]. In the case of Y100C variant, the replacement by a more polar amino acid at the end of TM2 segment (position 100) and the presence of a free sulfhydryl group in the amino acid C which increases its reactivity allowing the link to other molecules of C could have altered the conformation of HBsAg, affecting detection due to impaired recognition of epitopes and/or secretion due to defective insertion into the ER membrane and intracellular retention. However, HBsAg levels produced *in vitro* by Y100C variant were slightly higher (about 1.5-fold) than the levels produced by wild-type strain. Our findings indicated that Y100C mutation, which is potentially capable of altering protein conformation, reduce neither the production nor secretion of HBsAg.

## 5. Conclusions

Since previous studies have described that Y100C variation of the small S protein was frequently detected in the absence of HBsAg and was statistically associated with occult infection, the potential influence of this substitution in the occurrence of this peculiar type of chronic hepatitis B was investigated here. Our findings indicated that Y100C substitution alone did not negatively affect the detection and/or secretion of HBsAg. Further studies analyzing the complete genome of HBV strains with the Y100C substitution may elucidate whether this mutation affects HBV replication or if there are mutations in other HBV genomic region that could explain the association of these variants with occult hepatitis B infection.
